# Supplementary material for: Patterns of Intron Gain and Loss in Fungi
Source: PLoS Biol. 2004 Nov 30;2(12):e422. doi: 10.1371/journal.pbio.0020422 (PMC532390; doi:10.1371/journal.pbio.0020422)
Supplement: Table S1 — Also available at http://genes.mit.edu/NielsenEtAl/. (4.3 MB ZIP). [file pbio.0020422.st001.zip › NielsenEtAl/html/174.html]

AN7636.1.NCU06303.1.MG07773.1.FG01630.1


```
 CLUSTAL W (1.82) Multiple Sequence Alignments - Introns Inserted


Sequence 1: AN7636.1	353 aa
Sequence 2: FG01630.1	389 aa
Sequence 3: NCU06303.1	375 aa
Sequence 4: MG07773.1	362 aa
Alignment Length: 407 aa
Number Identitical Residues: 149 aa
Alignment Score (without introns) 7581


MG07773.1 	-------------------MAPEEKQVQRWQTVQDGVDKLWLEQAPVTTPGKDEVLVEIR
NCU06303.1	-------------------MSTQTKEVQQWHTFEDGFDNLTRGTAPMPTPGQDEVLVKIK
FG01630.1 	MTSFISSYLPFGKSETAANMATPET-NKQWTTGQDGLDKLQFGEGKVPQPKDGEVLVKIH
AN7636.1  	-------------------MSS----IQQWTTSLQGLDSLQSVEAPKPTAGPGEVLVRIS
          	                   *:.     ::* *  :*.*.*    .  . .  .****.* 

MG07773.1 	AVSLNYRDTE-~-------V1VNGLYKHHKSLQDKKPDPLTPCSDMCGVVTAVGP----D
NCU06303.1	AVSLNYRDTE-~-------V1TNGEYNHYEAYS-RRTTPLVPCSDALGIITAVGPLGPGA
FG01630.1 	AVGLNYRDTED1FIKLTAQV~INGDYNHHASV--QDTEALVPCSDMCGTVIQSSS-----
AN7636.1  	AVSLNYRDYE-~-------V1CCGLYGHHKSVS-QDKASLVPCSDMCGEIVEVGEG----
          	**.***** *         *   * * *: : . :   .*.****  * :   .      

MG07773.1 	LTVPWKVGDRVLSTFTQSHLSGQIKEEHMATGMGFPLDGVLQTQRVFPSTGLVKAPDSLS
NCU06303.1	SESAWKVGDRVLSTFCQSHLTGQITAKDLASALGKPLDGVLQTYRVFPSTGLVRVPEYLT
FG01630.1 	--PKLKVGTRVMSIFNQTHLTGQVVEADMASGLGLPLPGVLTEYRCFSADSLVTVPDYLS
AN7636.1  	-VTGWQKGDRVLSTFLPDHQTGQVVEKMMASGLGLPQPGVLAEYRAFPAHGLVRAPSYLS
          	     : * **:* *   * :**:    :*:.:* *  ***   * *.: .** .*. *:

MG07773.1 	DEEASCLPIAAVTAWMALFWQEPLREFP---------DLSGKVVVVQGTGGVSISGMQIA
NCU06303.1	DEEASCLPIAAVTAWMAIFGMEPFGTWATKASDDGKKPLQGKTVLLEGTGGVAIAGLQIA
FG01630.1 	DEEAACLPIAAVTAWMCINGMRPLGKPA-------DGGKKKESVLLQGTGGVAIAGLQIA
AN7636.1  	DEEAATLPIAAVTAWMSINGMRPKGQ---------NGGEG-EYILLQGTGGVSVSGLQIA
          	****: **********.:   .*            .     : ::::*****:::*:***

MG07773.1 	KAAGATT1IVTSSSDDKLQKAK-ALGADHLINYRTTPEWQDEVMKLTAGR-GADVIFECG
NCU06303.1	KAAGART~IITSSSDEKLRRAKEELGADHVINYKTTPDWEKKVMEITGENDGADIILETG
FG01630.1 	KAAGLNV~IITSSSDDKLERAKKDLGADTGINYRTHWEWQDPVMEATGGR-GADIIFETG
AN7636.1  	KASGAKT1IITSSSDDKLKQAK-ELGADYTINYRKTPNWEEEVLKLTDNH-GADIIIEVG
          	**:*  . *:*****:**.:**  ****  ***:.  :*:. *:: *  . ***:*:* *

MG07773.1 	GADTLHRSLQCVAFGGLVSCIGYLSGKQDKSVEG-RPHFNLLALSRNVTVKGLINGPRDR
NCU06303.1	GAGTLYKALDCIAFGGLISCIGYTAGKEDSGEGS-RMNVNVLALRRTVTLKGIINGPRDR
FG01630.1 	GARTLRKSFDSVAFGGLINCVGYLSGKEEEQDTGGKVGVNVLALRRNVTLKGILNGPKDR
AN7636.1  	GAGTLKKSFNCIAWGGLIDCVGYLSGKVDAPED--RTNVNLLALSRNVTLKGIINGPKDR
          	** ** ::::.:*:***:.*:** :** :      :  .*:*** *.**:**::***:**

MG07773.1 	FEDVVAFYDKHGIKPVVDRVFGFEEGRAALEYLMSGGHFGKVVVKISG--
NCU06303.1	FEEMCEFYQRHEIRPVVDKVFEFEEGKEAIKYLRSGSHFGKVVVKVE---
FG01630.1 	FEEMVSFYEKHAIHPVICKTFEFEEANKALEYLTSGQHFGKVVVKISKSG
AN7636.1  	FEEMIAFYEKHSIKPVVNKVFSFKDAPEAFKYLANGQHFGKIVIKIA---
          	**::  **::* *:**: :.* *::.  *::** .* ****:*:*:
```
